# Reduced estrogen signaling contributes to bone loss and cardiac dysfunction in interleukin‐10 knockout mice

**DOI:** 10.14814/phy2.15914

**Published:** 2024-01-12

**Authors:** Sanmi E. Alake, John Ice, Kara Robinson, Payton Price, Bethany Hatter, Karen Wozniak, Dingbo Lin, Winyoo Chowanadisai, Brenda J. Smith, Edralin A. Lucas

**Affiliations:** ^1^ Department of Nutritional Sciences Oklahoma State University Stillwater Oklahoma USA; ^2^ Department of Microbiology and Molecular Genetics Oklahoma State University Stillwater Oklahoma USA; ^3^ Department of Obstetrics and Gynecology Indiana School of Medicine Indianapolis Indiana USA; ^4^ Indiana Center for Musculoskeletal Health Indiana School of Medicine Indianapolis Indiana USA

**Keywords:** cardiovascular disease, estrogen, gut inflammation, inflammation, osteoporosis

## Abstract

Characterization of the interleukin (IL)‐10 knockout (KO) mouse with chronic gut inflammation, cardiovascular dysfunction, and bone loss suggests a critical role for this cytokine in interorgan communication within the gut, bone, and cardiovascular axis. We sought to understand the role of IL‐10 in the cross‐talk between these systems. Six‐week‐old IL‐10 KO mice and their wild type (WT) counterparts were maintained on a standard rodent diet for 3 or 6 months. Gene expression of proinflammatory markers and *Fgf23*, serum 17β‐estradiol (E2), and cardiac protein expression were assessed. Ileal *Il17a* and *Tnf* mRNA increased while *Il6* mRNA increased in the bone and heart by at least 2‐fold in IL‐10 KO mice. Bone *Dmp1* and *Phex* mRNA were repressed at 6 months in IL‐10 KO mice, resulting in increased *Fgf23* mRNA (~4‐fold) that contributed to increased fibrosis. In the IL‐10 KO mice, gut bacterial β‐glucuronidase activity and ovarian *Cyp19a1* mRNA were lower (*p* < 0.05), consistent with reduced serum E2 and reduced cardiac pNOS3 (Ser^1119^) in these mice. Treatment of ileal lymphocytes with E2 reduced gut inflammation in WT but not IL‐10 KO mice. In conclusion, our data suggest that diminished estrogen and defective bone mineralization increased FGF23 which contributed to cardiac fibrosis in the IL‐10 KO mouse.

## Graphical abstract

Unlike the WT (C57BL6) mouse, IL‐10 KO mouse exhibited tissue (gut, and consequently bone and cardiac) proinflammatory signature and reduced gut bacterial β‐glucuronidase activity. Proinflammatory cytokines favor increased bone production of FGF23, whose action (potentiated by reduced PHEX activity) could increase cardiac fibrosis. The reduced gut bacterial β‐glucuronidase activity decreases conjugated estrogen recycling into its active form, favoring reduced bone mass and cardiac phenotype in the IL‐10 KO mouse.

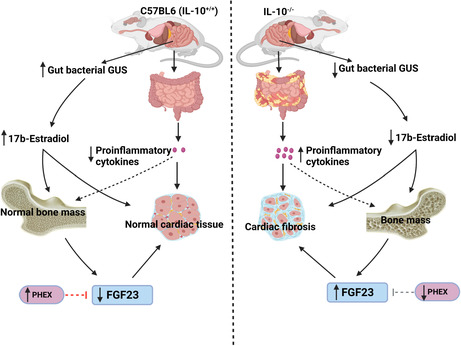

## 1 INTRODUCTION

Multidirectional interaction among organ systems is an integral aspect of the development and physiology of mammalian organisms. Factors, including hormones, cytokines, and chemokines, are produced by diverse cells in the body and participate in interorgan communication (Castillo‐Armengol et al., 2019). The mammalian gut is not an exception in the production of factors that mediate interorgan crosstalk for maintaining a healthy homeostatic state and for the normal functioning of several organ systems, including the musculoskeletal and cardiovascular systems (Hu et al., 2022; Lucas et al., 2018).

The gut microbiome encompasses a diverse array of gene batteries whose products (i.e., postbiotics) are continuously being characterized to participate in important aspects of hosts physiology, including gut (Martin‐Gallausiaux et al., 2021), cardiovascular (Razavi et al., 2019; Yang et al., 2019) and bone health (Lucas et al., 2018). More so, gut colonization with microbiota is critical for shaping the mucosal immune system and results in either a homeostatic or abnormal immune reactivity (Al Bander et al., 2020; Brandsma et al., 2019). Importantly, the mammalian gut has evolved to guard against the translocation of unwanted substances, including toxins and pathogens, into the systemic circulation. In addition, ~70% of our immune cells reside in the gut, functioning in antigen sampling, and producing pathogen‐specific immune responses (Wiertsema et al., 2021). Aging‐induced alterations in the mammalian gut microbiome and immune cell function increase chronic inflammation (i.e., inflammaging) in the gut and increase the risk of chronic diseases, including cardiovascular disorders (CVDs) and osteoporosis (Bosco & Noti, 2021; Fasano, 2020; Gibon et al., 2017; Li et al., 2019; Liberale et al., 2022).

Interleukin (IL)‐10 is a well‐known pleiotropic anti‐inflammatory cytokine (Sabat et al., 2010). The partial or total loss of this cytokine, as in the IL‐10 knockout (KO) mouse, models inflammaging and is associated with spontaneous enterocolitis (Kühn et al., 1993), osteopenia and increased bone fragility (Dresner‐Pollak et al., 2004), and cardiac events including thrombosis, (Caligiuri et al., 2003), atherosclerosis (Mallat et al., 1999), arterial stiffness and cardiac dysfunction (Sikka et al., 2013). Additionally, a role for inflammatory response mediators, including interleukin (IL)‐6 and tumor necrosis factor (TNF) in CVDs and bone resorption have been reported (Epsley et al., 2021; Willerson & Ridker, 2004). Research also supports an anti‐inflammatory role for certain postbiotics such as butyrate (Säemann et al., 2000). However, other roles of gut microbiota in reducing inflammation and the crosstalk between the gut, bone, and cardiovascular system are still poorly understood.

Fibroblast growth factor (FGF)‐23 and estrogen are hormones derived mainly from the bone and ovaries (testes in males), respectively, and are important regulators of bone metabolism and cardiovascular outcomes (Lu & Hu, 2017; Quarles, 2012). Whereas inflammation, directly and indirectly, increases phosphaturic FGF23 (Francis & David, 2016), an anti‐inflammatory role has been mainly attributed to estrogen (Chakrabarti et al., 2008; Josefsson et al., 1992), a hormone that prevents bone loss (Kameda et al., 1997; Nakamura et al., 2007) and reduces vascular injuries and atherosclerosis (Arnal et al., 2010; Meng et al., 2021; Xing et al., 2009). Egli‐Spichtig et al. (2019) reported increased serum intact FGF23 (iFGF23) in IL‐10 KO mice at 12–14 weeks of age. However, the clinical significance of this finding in relation to skeletal and cardiovascular outcomes has not been investigated.

An important aspect of estrogen metabolism is the recycling of its conjugated form by gut bacterial, β‐glucuronidase (Baker et al., 2017). While aging causes an alteration in the metabolic signature of the gut microbiome, the role of inflammation in estrogen recycling and signaling has not been investigated. We aimed to examine the crosstalk between the gut, bone, and cardiovascular system in an IL‐10 KO mouse model. We hypothesized that the anti‐inflammatory cytokine IL‐10 is critical for fine‐tuning gut‐microbial derived factors, including deconjugated estrogen and inflammatory stimuli, which in turn play critical roles in normal bone remodeling and cardiovascular function.

## 2 METHODS

### 2.1 Animals

Animals were maintained at the Oklahoma State University Laboratory Animal Research facility under humidity‐ and temperature‐controlled conditions and a 12‐h light–12‐h dark cycle.

Six‐week‐old male and female IL‐10 KO mice (KO) and their wild type (WT) counterpart of C57BL/6 background were (Jackson Laboratories, Bar Harbor, ME) maintained on a semi‐purified rodent growth diet (AIN‐93G) for the first 3 months following weaning and then changed to maintenance diet (AIN‐93M) for the final 3 months of the study. Mice had access to food and water ad libitum. Weekly food intake and body weight were monitored during the study period.

### 2.2 Sample collection and tissue processing

Fecal samples were collected per cage and stored at −80°C after 3 and 6 months on the AIN‐93 diet. At the end of each time point, mice were fasted for ~3 h and anesthetized with a ketamine and xylazine cocktail (100 mg and 10 mg, kg^−1^ body weight, respectively). Whole‐body dual‐energy x‐ray absorptiometry (DXA) scans were performed to determine bone mineral content (BMC) and bone mineral density (BMD) using a whole‐body densitometer (Piximus; GE Medical System Lunar, Madison, WI). Serum was processed from blood collected from the carotid artery and stored at −80°C. The heart, aorta, and white adipose tissue (WAT) were excised and snap‐frozen in liquid nitrogen. Lamina propria (LP) was collected from saline‐flushed ileum and stored in RNALater. A femur from each mouse was flushed with ice cold PBS and the marrow‐free bone was snap‐frozen and cryo‐stored until analyses.

### 2.3 Serum 17β‐estradiol and cecal content β‐glucuronidase activity

Serum unconjugated 17β‐estradiol (E2) was assessed using an ELISA kit (Biovision; Milpitas, CA) following the manufacturer's instructions.

Bacterial β‐glucuronidase activity in cecal content was assessed with slight modifications to previous protocols (Flores et al., 2012; Walsh et al., 2020). Briefly, approximately 300 mg of frozen cecal content was homogenized in 1 mL ice‐cold PBS. Lysates were centrifuged at 2000 rpm for 5 min at 4°C and the supernatant was further centrifuged at 10,000 rpm for 20 min. Enzyme activity was assessed in the supernatant by mixing 25 μL of supernatant, 50 μL PBS, and 50 μL 4‐nitrophenyl‐β‐d‐glucuronide (1 mM, dissolved in PBS; Sigma‐Aldrich, St. Louis, MO # 10344‐94‐2). The resulting mixture was incubated at 37°C for 15 min, followed by the addition of 125 μL sodium hydroxide (NaOH, 0.5 N, ThermoFisher #BP359) to stop the reaction. The absorbance was read at 405 nm on a Biotek HTX microplate reader. Enzymatic concentrations were extrapolated from a standard curve of a serially diluted pure enzyme purchased from Sigma‐Aldrich (# G7646). Enzyme activities were normalized by the total protein content in each sample.

### 2.4 X‐ray micro‐computed tomography (μCT) analyses

μCT analyses (μCT40; SCANCO Medical, Brüttisellen, Switzerland) were performed on the lumbar vertebral body (L6), distal femur metaphysis, and the femur mid‐diaphysis to determine alterations in trabecular and cortical bone microarchitecture. Femur specimens were scanned at a high resolution of 2048 × 2048 pixels. Analysis of the distal femur was performed on a volume of interest (VOI) that included a region of secondary spongiosa that was 60 μm from the growth plate and extended in the proximal direction 600 μm (100 images). A 180 μm (30 images) VOI at the mid‐point of the femur was used for cortical bone analysis. Scanning of the vertebra was performed at medium resolution or 1024 × 1024 pixels. The VOI included a region of secondary spongiosa that was approximately 2.56 mm (160 images × 16 μm ea). Analysis of all specimens was performed at the threshold of 360, a sigma of 1.2, and a support setting of 2. The trabecular analysis for the distal femur metaphysis and vertebral body included the following: relative bone volume (BV/TV), trabecular number (TbN), trabecular thickness (TbTh), trabecular separation (TbSp), connectivity density (ConnDens), structural model index (SMI), apparent density, material density, and degree of anisotropy. Cortical analyses of the tibial mid‐diaphysis included cortical thickness, cortical area, medullary area, and porosity.

### 2.5 Gene expression analysis

Total RNA was isolated from ileum LP, WAT, heart, aorta, ovary, bone marrow, and femur by rupturing tissues in Trizol RNA isolation reagent (ThermoFisher, Waltham, MA, #10‐296‐028) and precipitating chloroform‐separated clear phase in isopropanol as earlier described (Peirson & Butler, 2007). cDNA was synthesized following a standardized protocol (Peterson & Freeman, 2009), and the relative abundance of genes was determined by quantitative reverse transcriptase‐polymerase chain reaction (qRT‐PCR) using SYBR green chemistry (Applied Biosystems, #A25742) on a CFX Opus RT‐PCR System (Bio‐Rad, Hercules, CA). Gene targets included interleukin (*Il*)*‐17a*, *Il1b*, *Il6*, *Tnf*, and *Tgfb*, estrogen receptor (*Esr*)*‐1* and *2*, tight junction protein 1 (*Tjp1*) and occludin (*Ocln*) in the ileum. *Il17a*, *Tnf*, *Il6*, *Il1b*, intercellular and vascular cell adhesion molecule 1 (*Icam1* and *Vcam1*), *Esr1*, adhesion G protein‐coupled receptor E1 (*Adgre1*), and arginase 1 (*Arg1*) were assessed in cardiac tissues. Dentin matrix protein 1 (*Dmp1*), phosphate regulating endopeptidase homolog X‐linked (*Phex*), *Fgf23*, *Il6*, wingless‐type MMTV integration site family member 10B (*Wnt10b*), osteoprotegerin (*Opg*), *Rankl*, collagen type 1 alpha 1 (*Col1a1*), bone gamma‐carboxyglutamate protein 2 (*Bglap2*), secreted phosphoprotein 1 (*Spp1*), and sclerostin (*Sost*) were assessed in the bone tissue. *Esr 1* and *2*, *Cyp19a1* (aromatase), and *Cyp11a1* were assessed in the WAT and ovary. The data were analyzed using the 2−ΔΔCt method (Schmittgen & Livak, 2008), with glyceraldehyde‐3‐phosphate dehydrogenase (*Gapdh*) serving as the invariant control. Primers were either derived from previous reports in the literature or designed in our laboratory and then validated in our hands before gene expression analyses were performed. Table S1 contains the list of primer sequences.

### 2.6 Immunoblotting

Total protein was extracted and quantified from heart tissue using the radioimmunoprecipitation assay (RIPA) buffer containing 0.5% protease and phosphatase inhibitor cocktails (Sigma‐Aldrich, #P8340 #P0044). Proteins were denatured and separated on polyacrylamide gels (BioRad, #4561093EDU) using SDS‐PAGE before transfer on PVDF membranes (ThermoScientific, Waltham, MA, #PI88585) as previously described (Ojo et al., 2021). Membranes were incubated overnight at 4°C in primary antibodies: (p‐eNOS Ser1119 [ThermoFisher, #PIPA564613]; NOS3 [ThermoFisher, #4904]; p‐IKB [Santa Cruz Biotechnology, Dallas, TX, #SC‐8404]; PI3K [Santa Cruz Biotechnology, #SC‐365290]; IKB [Santa Cruz Biotechnology, #SC‐1643]; p‐NFkBp65 Ser536 [#sc‐136548, Santa Cruz Biotechnology]; NFkBp65 [Santa Cruz Biotechnology, #SC‐8008]; and GAPDH [#60004–1, Proteintech, Rosemont IL]) diluted in 5% bovine serum albumin (BSA). Membranes were washed in PBS and incubated for 1 h in anti‐rabbit (Cell Signaling Technology, Danvers, MA #7074) or anti‐mouse (Cell Signaling Technology, #7076) IgG HRP‐linked antibody diluted in 5% milk solution. Proteins were detected using the SuperSignal West Femto Maximum Sensitivity Substrate (ThermoScientific, #34095). Band signals were captured with the ChemiDoc imaging system (BioRad) and quantified with the Image J software, v 1.8.0.

### 2.7 Cell culture experiments

Intestinal lymphocytes were isolated from the ileum of 14 month‐old female KO and WT mice (*n* = 4/strain) maintained on standard chow as previously described with minor modifications (Sheridan & Lefrançois, 2012). Briefly, tissues were flushed with RPMI medium (ThermoFisher, #61‐870‐036) supplemented with 2% FBS and 1 mM dithiothreitol (DTT, Sigma‐Aldrich, #D9779). Ileal samples were opened longitudinally and incubated at room temperature in Hanks' balanced salt solution (HBSS) with 2 mM EDTA. Epithelia‐free samples were digested with collagenase type VIII (Sigma‐Aldrich #C2139) and filtered through a 70 μm cell strainer. Lymphocytes were subsequently separated, by differential centrifugation, as an interphase between Percoll gradients (40% and 80%, Sigma‐Aldrich, #GE17‐0891‐01). Cells were collected, washed twice, and incubated for 40 min in a 10% FBS‐supplemented RPMI with or without E2 (100 nM, Sigma‐Aldrich, #E2758).

For 3‐dimensional cultures, intestinal crypts were isolated from the ileum of 14 month‐old female KO and WT mice and developed into organoids as previously described (Sato et al., 2009) with minor modifications. Briefly, the small intestine was excised, opened longitudinally and gently flushed with cold PBS. Tissues were cut into small pieces (~2 mm) and gently washed several times in PBS until the supernatant became clear (~20 times). Tissues were digested at room temperature in gentle cell dissociation reagent (Stemcell Technologies, Cambridge, MA, #07174), resuspended in cold 0.1% BSA‐containing PBS, pipetted up and down three times, and allowed to settle under gravity. The wash, digestion, and dissociation step were repeated three times. Crypt‐enriched fractions derived by passing supernatant from the fourth digestion through a 70 μm strainer was centrifuged and resuspended in complete mouse intesticult organoid growth medium (Stemcell Technologies, #06005) and geltrex basement membrane matrix (Thermofisher, #A1413302) before plating in triplicate in a 24‐well plate. Once solidified, organoids were formed in complete mouse intesticult organoid growth medium treated with or without E2 (100 nM).

#### 2.7.1 Statistical analyses

Data were evaluated for conformity to normal distribution. Two‐group comparisons of IL‐10 KO and their sex‐matched WT control were performed for all outcome variables using the independent Student *t*‐test in SAS version 9.4 (SAS Institute, Cary, NC). Data are presented as mean ± standard deviation (SD), and *p* value <0.05 was considered statistically significant.

## 3 RESULTS

### 3.1 Gut inflammation increased in IL‐10 KO mice

**FIGURE 1:**
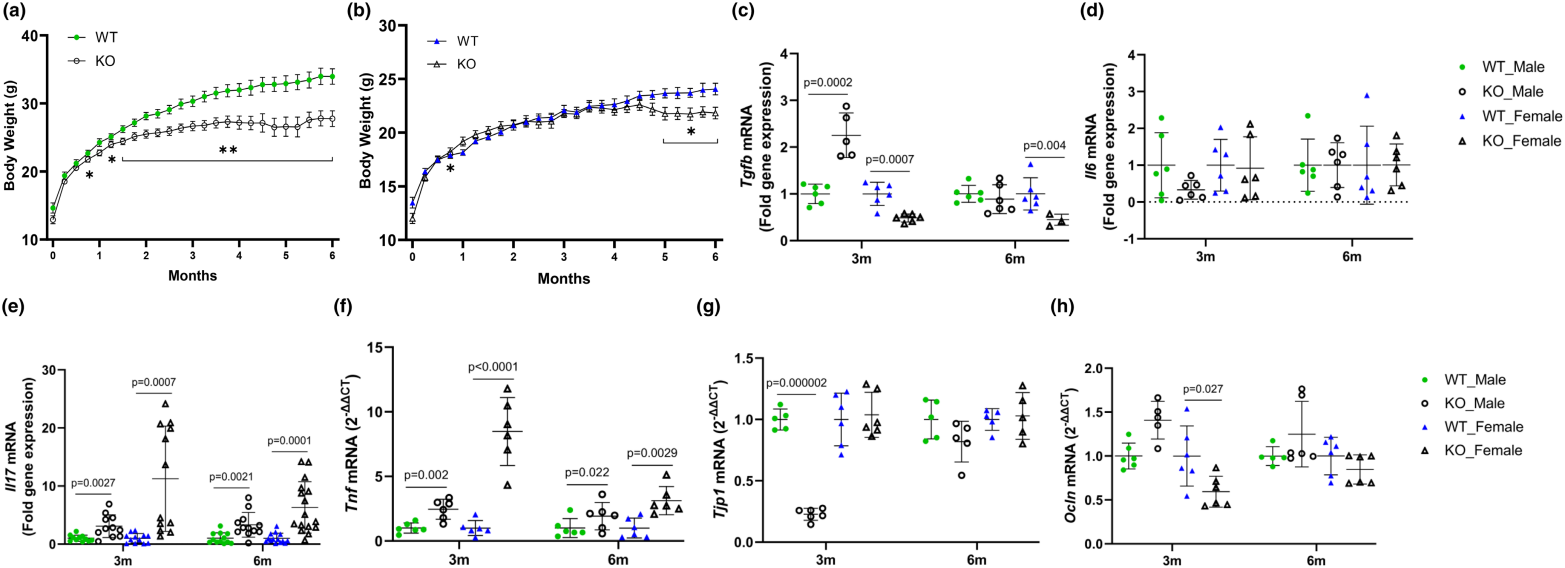
Weekly body weight of male (a) and female (b) mice, and ileum gene expression of inflammatory cytokines, *Tgfb* (c), *Il6* (d), *Il17a* (e), *Tnf* (f), and tight junctions, *Tjp1* (g), and *Ocln* (h) in WT mice and IL‐10 KO (KO) mice fed a semi‐purified diet for 3 or 6 months. Values are mean ± SD, *n* = 12–16 mice/group for a and b, *n* = 6 mice/group for c and d, *n* = 12–16 mice/group for e, and *n* = 6 mice/group for f–h. Based on data analysis using Student's *t*‐test.

Compared to their WT gender‐matched counterparts, body weight was significantly lower (*p* < 0.01) in KO male mice from Week 6 to 24 (Figure 1a). In contrast, the female KO mice maintained similar body weight as the WT counterparts until Week 19, and then exhibited reduced body weight (*p* < 0.05) until the end of the experiment (Figure 1b). The reduced body weight in the male and female KO mice corresponds with lower food intake (Figure S1a) and most likely be due to the increase in gut inflammation observed in these mice.

We next examined the relative mRNA abundance of inflammatory cytokines in the ileal LP as an index of inflammation. The anti‐inflammatory cytokine, *Tgfb*, increased in KO male at 3 months (*p* = 0.00014), but decreased in KO female at both time points (*p* < 0.01, Figure 1c). There were no significant differences in *Il6* gene expressions for either sex compared with WT controls (Figure 1d). However, *Il17* and *Tnf* mRNA increased significantly in both male (*p* < 0.05) and female KO (*p* < 0.01) mice at both timepoints compared to their WT sex‐matched counterparts (Figure 1e,f). As inflammation is crucial to compromising intestinal integrity, we examined tight junction gene expression. At the 3 month timepoint, ileal expression of *Tjp1* was lowered in male (*p* < 0.0001) and *Ocln* in female (*p* = 0.027) KO compared to their WT counterpart (Figure 1g). However, by the 6 month timepoint, there were no differences in the expression of *Tjp1* and *Ocln* (Figure 1g,h).

### 3.2 Increased inflammation and FGF23 are associated with osteopenia in IL‐10 KO mice

**FIGURE 2:**
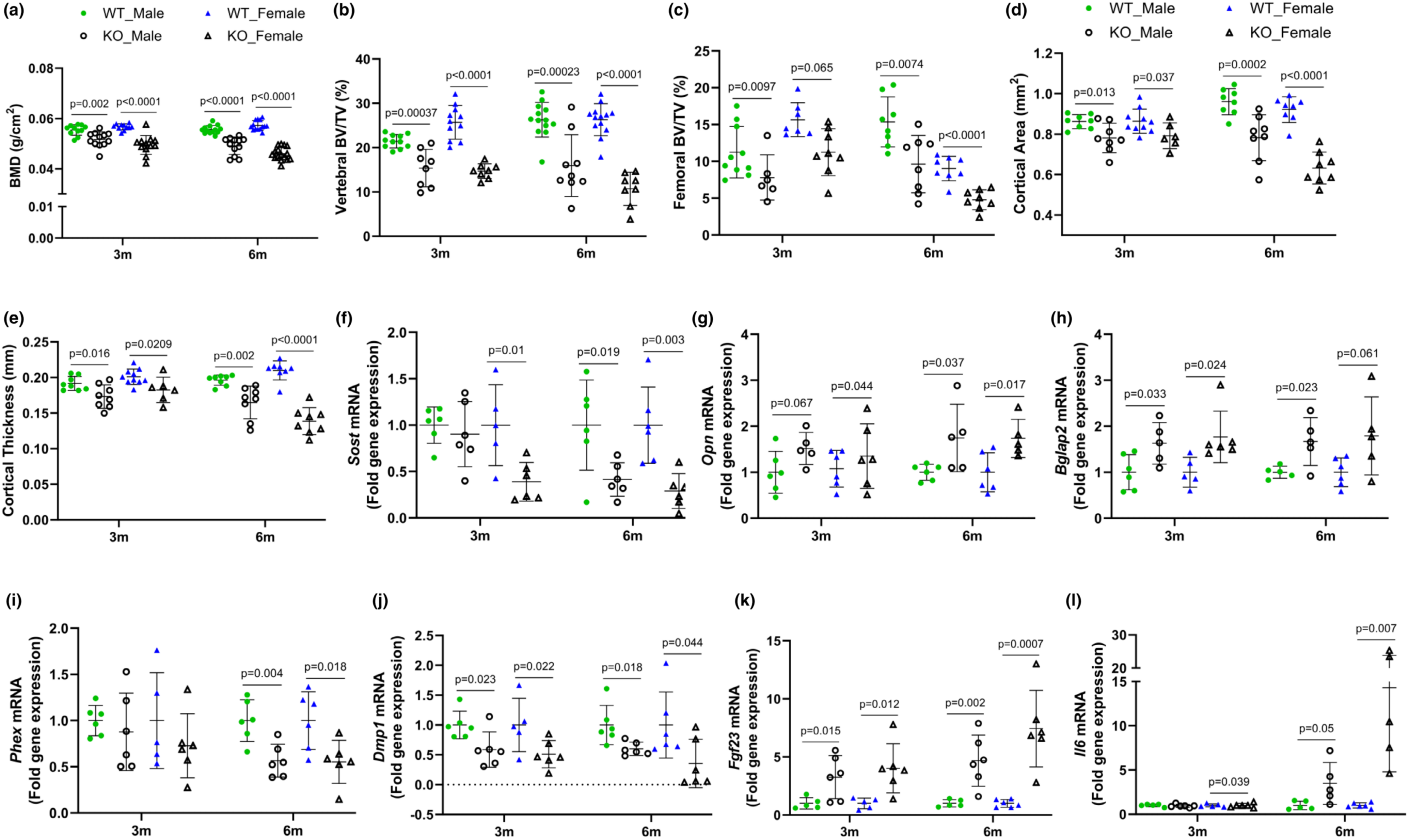
Skeletal response of male and female WT and IL‐10 KO (KO) mice fed a semi‐purified diet for 3 or 6 months: whole body bone mineral density, BMD (a), trabecular bone volume/total volume (BV/TV) of the lumbar vertebra body (b) and distal femur metaphysis (c), femur cortical area (d) and thickness (e), and gene expression of *Sost* (f), *Opn* (g), *Bglap2* (h) *Phex* (i) *Dmp1* (j), *Fgf23* (k), and *Il6* (l). Values are mean ± SD, *n* = 12–16 mice/group for a, *n*‐8–13 mice/group for b–e, *n* = 6 mice/group for f–l. Based on data analysis using Student's *t*‐test.

To confirm the effects of *Il10* KO on bone parameters, we performed a whole‐body DXA scan. At 3 and 6 months, both male and female KO mice had lower (*p* < 0.05) BMD than their WT counterparts (Figure 2a). The significant loss of BMC in the KO male mice (Figure S1b) is primarily responsible for the reduced BMD since bone mineral area (BMA) was either somewhat reduced or not affected in these mice (Figure S1c). However, both BMC and BMA were significantly reduced in female KO mice at both time points (Figure S1b,c).

Next, we evaluated the alterations in trabecular bone in the distal femur metaphysis and vertebral body using microCT. At the 3 month timepoint, vertebral BV/TV was lower in male and female KO mice by ~20% and 40% compared to their respective WT controls (Figure 2b). By 6 months, the magnitude of the difference in vertebral bone of the male and female KO compared to WT controls was even greater, with an ~40% and 60% decline, respectively. The lower vertebral BV/TV was associated with significant reductions (*p* < 0.05) in TbN and TbTh with a corresponding increase in TbSp in KO mice versus WT controls (Figure S2a–c). Similarly, the reduction in BV/TV within the distal femur metaphysis of male KO mice was ~30%–33% at 3 and 6 months, whereas female KO mice exhibited a trend at 3 months (*p* = 0.065; ~27% reduction) and a statistically significant reduction (*p* < 0.0001; 44%) at 6 months compared to WT counterpart (Figure 2c). The reduced femoral trabecular bone volume was attributed to a corresponding lower TbTh in KO mice in both sexes at both timepoints (Figure S2d). Conversely, there were no statistically significant differences in the femur TbN and TbSp for the strains of mice that were compared (Figure S2e,f).

Cortical bone was also compromised in the IL‐10 KO mice, with ~10% reduction (*p* < 0.05) in femoral cortical area and thickness at the 3 month timepoint, and ~20% reduction (*p* < 0.01) in these parameters at the 6 month timepoint (Figure 2d,e). At the transcriptional level, the relative abundance of *Sost*, the Wnt signaling inhibitor, was lower in the femur (*p* < 0.05) of male KO mice at 6 months and at both timepoints in the female KO mice compared to their WT controls (Figure 2f). Interestingly, *Wnt10b* mRNA was also repressed (*p* < 0.05) in male KO at both timepoints and upregulated in female KO mice at 6 months versus WT mice (Figure S2g). Assessment of the mRNA expression of extracellular matrix proteins revealed similar *Col1a1* mRNA level in both WT and KO mice (Figure S2h), whereas *Opn* and *Bglap2* had at least ~1.5‐fold increased expression in KO mice compared to gender‐matched WT mice at both the 3 and 6 month timepoints (Figure 2g,h). Next, we assessed the gene expression of receptor activator of nuclear factor kappa B ligand (RANKL), which regulates the catabolic activity of bone and OPG, a decoy receptor that inhibits RANKL‐RANK interaction. The relative abundance of *RankL* was reduced (Figure S2i) with a corresponding lower *Opg* gene expression (*p* < 0.05) in both male and female KO compared to WT control mice at both timepoints (Figure S2j). These data support an attempt to down‐regulate osteolytic activity in the bone of the KO mice.

We next examined gene expression of the endopeptidase, phosphate‐regulating endopeptidase X‐linked (PHEX) and its sibling protein, dentine matrix acidic phosphoprotein 1 (DMP1), both produced by osteocytes and participating in bone mineralization (Schaffler & Kennedy, 2012). *Phex* was significantly reduced at 6 months and *Dmp1* was reduced at both timepoints in KO mice compared with sex‐matched WT control (Figure 2i,j). DMP1 and Phex are involved in the regulation of *Fgf23* (Martin et al., 2011). As expected, *Fgf23* mRNA increased (*p* < 0.05) with reducing *Phex* and *Dmp1* in KO mice at both timepoints compared to their WT control (Figure 2k). Importantly, *Il6* mRNA tended to increase at 6 months in male KO, and was significantly increased (*p* < 0.05) at both timepoints in female KO mice compared to WT control (Figure 2l).

### 3.3 Loss of IL‐10 is associated with increased cardiac inflammation and fibrosis in mice

**FIGURE 3:**
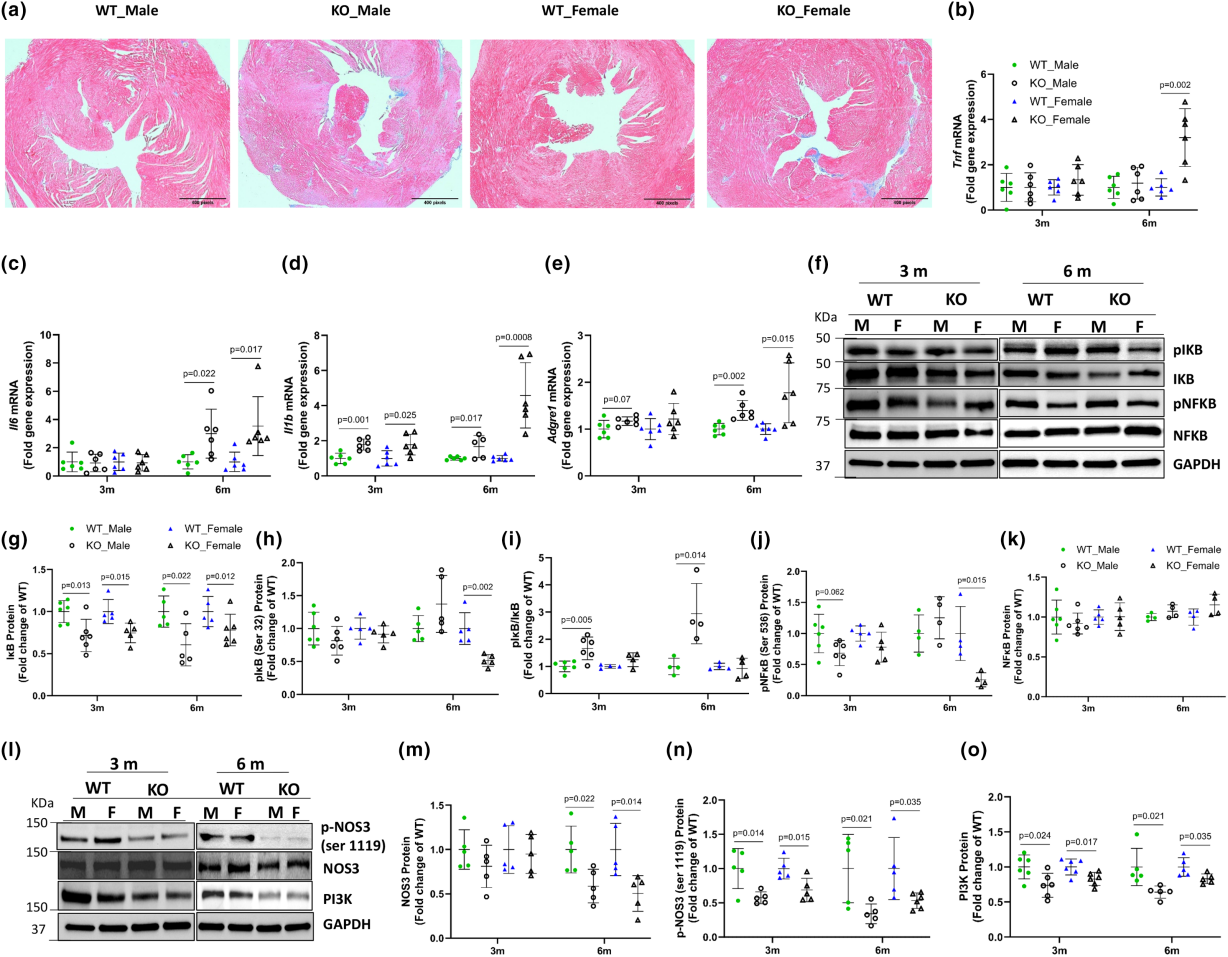
Representative trichrome staining of fixed heart tissue, with blue coloration indicating fibrosis (a), cardiac gene expression of inflammatory markers: *Tnf* (b), *Il6* (c), *Il1b* (d), and the macrophage marker *Adgre1* (e). Representative blots (f), showing cardiac protein expression of: IκB (g), phosphorylated IκB (h), Ratio of pIκB to total IκB (i), phosphorylated NFκB (j), and NFκB (k). Also, representative blots (l) for cardiac protein expression of NOS3 (m), phosphorylated NOS3 (n), and PI3K (o) in WT mice and IL‐10 KO (KO) mice fed a semi‐purified diet for 3 or 6 months. Values are mean ± SD, *n* = 6 mice/group for a–e, *n* = 4–5 mice/group for f–o. Student's *t*‐test.

Motivated by the reported role of excessive FGF23 in inducing left ventricular hypertrophy, cardiac fibrosis, and vascular calcification (Böckmann et al., 2019; Faul et al., 2011; Hao et al., 2016; Jimbo et al., 2014), we next examined transverse cross‐section of the heart for histological changes. Masson's trichrome staining revealed increased fibrosis in the cardiac tissue of KO mice at 6 months compared with WT control mice (Figure 3a). Cardiac gene expression of the inflammatory marker, *Tnf*, only reached statistical significance at 6 months in female KO mice (Figure 3b). Similarly, the *Il6* transcript was significantly elevated (*p* < 0.05) in the KO mice at the 6 months timepoint in both sexes compared to their respective WT control (Figure 3c). In contrast, the relative abundance of *Il1b* expression was higher (*p* < 0.05) in the cardiac tissue of KO mice at both timepoints (Figure 3d). Gene expression of adhesion molecules, including, vascular cell adhesion protein 1 (VCAM‐1) and intercellular adhesion molecule‐1 (ICAM‐1) was increased (*p* < 0.05) in the cardiac tissue of female KO mice at the 6 month timepoint. (Figure S3a,b). Gene expression of the receptor *Adgre1*, a macrophage marker, increased significantly in the cardiac tissues of KO mice at 6 months (Figure 3e). The M2 macrophage marker, *Arg1*, tended to be reduced (0.05 < *p* < 0.1) in female KO mice at 3 months, but was reduced (*p* < 0.05) in the cardiac tissue of male KO mice at both timepoints compared with WT control (Figure S3c).

Next, we assessed the protein expression/activation of the redox‐sensitive transcription factor, NF‐κB and key components of its signaling pathway. Inhibitor of nuclear factor kappa B (IκB), which prevents the nuclear translocation of NF‐κB, was reduced (*p* < 0.05) in both sexes of KO mice at the 3 and 6 month timepoints (Figure 3f,g), with female KO exhibiting reduced phosphorylation (*p* < 0.01) of IκB at 6 months (Figure 3f,h). The ratio of phosphorylated to total IκB increased (*p* < 0.05) at both time points in male KO (Figure 3f,i). The reduced phosphorylation of IκB at 6 months in female KO mice correlates with a reduced (*p* < 0.05) phospho‐NFκB (Ser^536^) in these mice at this timepoint (Figure 3f,j,k).

We further assessed the protein expression of endothelial nitric oxide synthase (NOS)‐3, whose Ser^1119^ phosphorylation is critical in nitric oxide‐mediated vasodilation. KO mice had a reduced expression of NOS3 (*p* < 0.05) at the 6 month timepoint (Figure 3l,m). Reduced activation of this enzyme as indicated by its reduced Ser^1119^ phosphorylation coincided with a reduction in phosphoinositide 3‐kinase (PI3K), which lies downstream of the NOS3 signaling, at both timepoints compared with respective WT control (Figure 3m–o).

### 3.4 Loss of IL‐10 is associated with reduced estrogen signaling in mice

**FIGURE 4:**
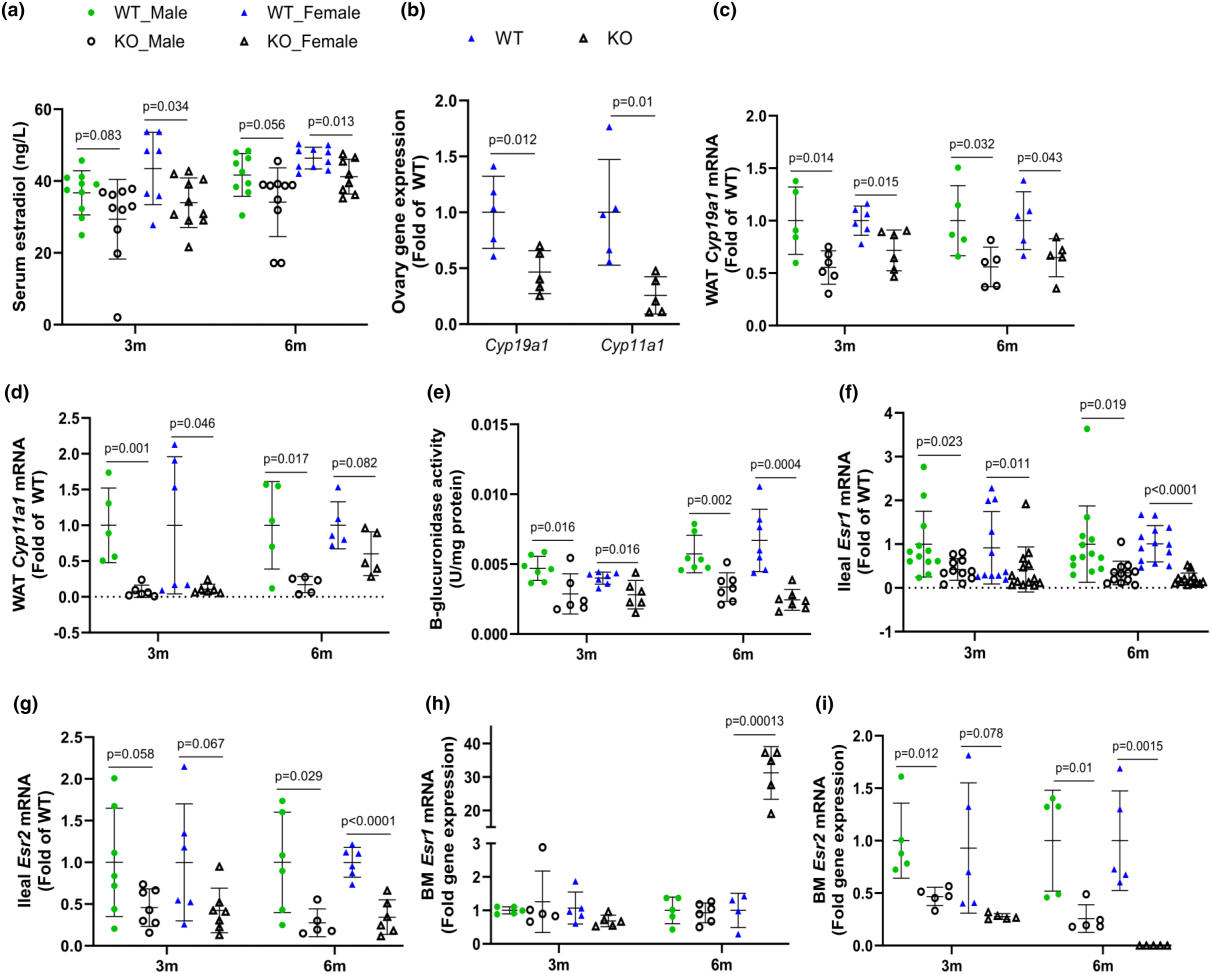
Serum 17β‐estradiol (a). Gene expression of *Cyp19a1* and *Cyp11a1* in the ovary (b), and white adipose (WAT; c and d, respectively). β‐glucuronidase enzyme activity in the cecal contents (e), and estrogen receptor gene expression in the ileum, Esr1 (f) and Esr2 (g) and bone marrow (h and i) in WT mice and IL‐10 KO (KO) mice fed a semi‐purified diet for 3 or 6 months. Values are mean ± SD, *n* = 8–10 mice/group for a, *n* = 5 mice/group for b, *n* = 6 mice/group for c and d, *n* = 7 mice/group for e, *n* = 5–6 mice/group for f–i. Based on data analysis using Student's *t*‐test.

Considering the role of estrogen as an activator of NOS3 (Chen et al., 1999) and the role of this hormone in reducing inflammation (Liberale et al., 2022), cardiac dysfunction, and bone loss (Arnal et al., 2010; Meng et al., 2021; Xing et al., 2009), we compared serum 17β‐estradiol (E2) in KO mice with WT control mice. Serum E2 was lower (*p* < 0.05) in female KO mice and tended to be reduced (0.05 < *p* < 0.1) in male KO mice at both timepoints compared with WT controls (Figure 4a). Following this serendipitous finding of reduced E2 in the KO mice, we assessed estrogen synthesis in the ovaries of 14 month‐old mice. Concurrently, mRNA levels of *Cyp11a1* and *Cyp19a1*, which are involved in estrogen synthesis, were significantly reduced in the ovaries of 14 month‐old KO mice compared with WT counterparts of the same age (Figure 4b). Similar reductions in mRNA levels were also observed in the WAT, another source of endogenous E2, of KO mice at the 3 and 6 month timepoints (Figure 4c,d). The activity of microbial β‐glucuronidase within the gut, which deconjugates estrogen in the intestinal lumen, was reduced at both time points in KO mice (Figure 4e). Estrogen could mediate its effects in several ways, one of which is binding to receptors, including ESR1 and ESR2, to mediate a membrane response or genomic response (Heldring et al., 2007). *Esr2* was not expressed in the heart (data not shown), whereas *Esr1* mRNA was not different between groups compared at both timepoints (Figure S4a). Contrarily, ileal and WAT *Esr1* mRNA were significantly reduced (*p* < 0.05) in KO mice at both timepoints (Figure 4f; Figure S4b), while the reduction in ileal *Esr2* mRNA only reached statistical significance in the ileum tissue at the 6 month timepoint (Figure 4g). *Esr1* was highly expressed at the 6 month timepoint in the bone marrow of female KO, while *Esr2* was repressed within the bone marrow at both timepoints in KO mice compared to WT control mice (Figure 4h,i).

**FIGURE 5:**
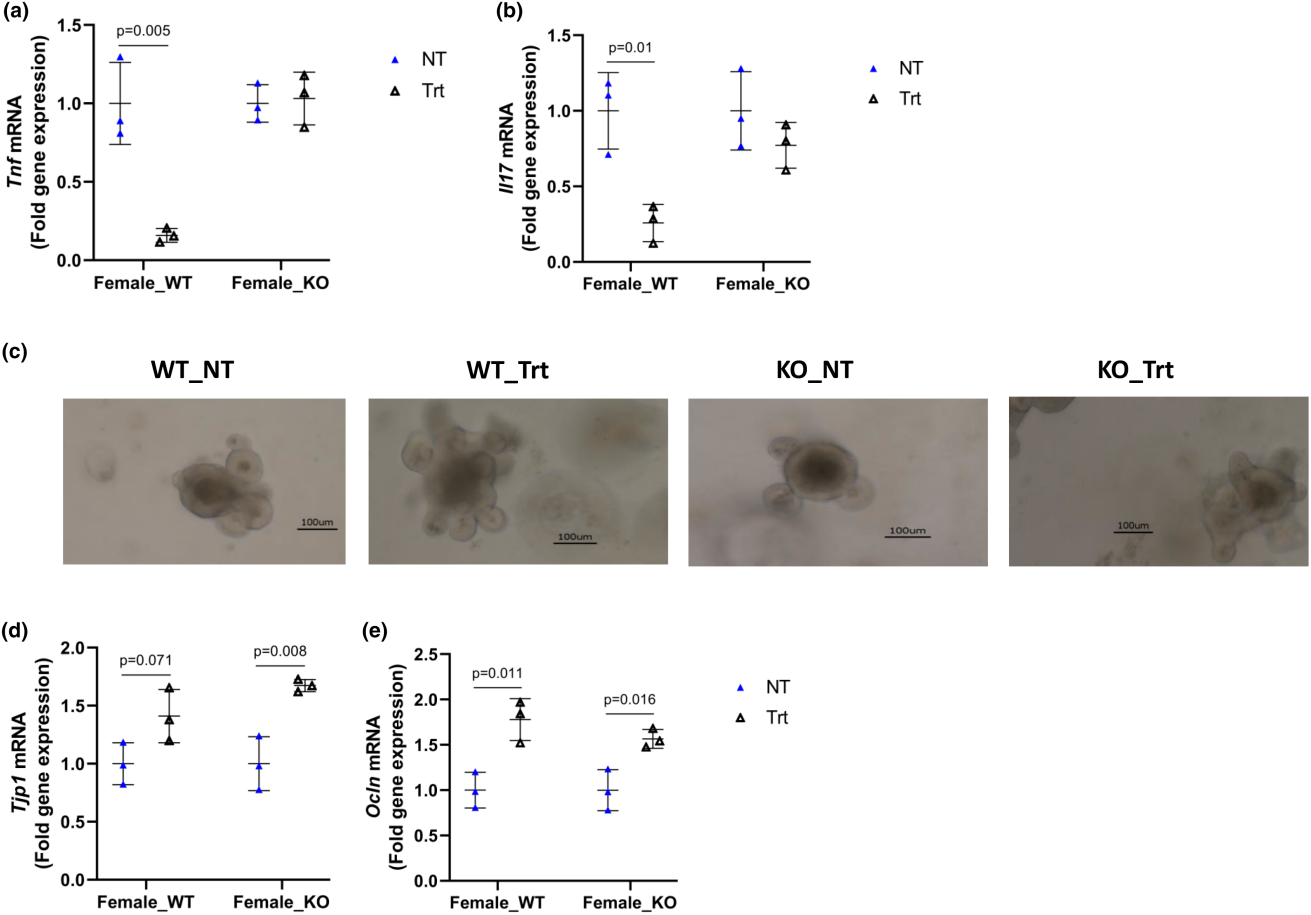
Gene expression of proinflammatory cytokines in E2‐treated (100 nM) ileal‐derived lymphocytes isolated from 14 month‐old‐female KO and WT mice: *Tnf* (a), and *Il17* (b). Representative image of intestinal organoids, Day 6, developed from crypt cells derived from 14 month‐old female KO and WT mice, with or without E2 treatment (c). Gene expression of tight junctions in E2‐treated intestinal organoids: *Tjp1* (d), and *Ocln* (e). Values are mean ± SD, *n* = 3 mice/group for a, b, *n* = 3 mice/group and replicate = 3/mouse for c–e. Based on data analysis using Student's *t*‐test.

To further confirm if IL‐10 is required for estrogen to modulate gut inflammation, lymphocytes from the ileum of 14 month‐old‐female WT and KO mice were isolated and treated with conditioned media with or without E2 (100 nM). E2 treatment lowered lymphocytic *Tnf* and *Il17* mRNA in WT, but not in the KO mice (Figure 5a,b). We further assessed the effect of E2 treatment (100 nM) on intestinal crypt‐derived organoids from female KO and WT mice. E2‐treated organoids were faster in developing complex structures (crypt domain) in both WT and KO groups (Figure 5c). The mRNA expression of *Vil1* and *Alpi*, which are highly expressed in the intestine, were not affected by E2 treatment in both organoid from WT and KO mice (Figure S5a,b). In contrast, E2 treatment increased gene expression of *Tjp1* in the KO‐derived organoid (Figure 5d), and *Ocln* in organoid from both WT and KO mice (Figure 5e). These data support an estrogenic effect on the enterocytes and not the lymphocytes of old IL‐10 KO mice.

## 4 DISCUSSION

In addition to its role in balancing gut immune reactivity, IL‐10 plays a critical role in interorgan communication (Madsen et al., 1997; Miyoshi et al., 2021; Oshima et al., 2001). The mammalian gut participates in several interorgan communications in the mammalian system (Forkosh & Ilan, 2019; Tripathi et al., 2018; Zaiss et al., 2019). Aging‐associated alterations in these interorgan communications contribute to chronic diseases, including cardiovascular and musculoskeletal disorders. Using the IL‐10 KO frail mouse model (Walston et al., 2008), this study investigated the role of IL‐10 in mediating interplay between the gastrointestinal, skeletal, and cardiovascular systems. We reported a proinflammatory signature in the gut, bone loss and higher bone *Fgf23* mRNA, lower serum estrogen, and increased cardiac fibrosis in the absence of IL‐10.

The highly expressed *Il17a* and *Tnfa* mRNA in the gut of the IL‐10 KO mice is expected, as IL‐10 suppresses both Th1 and Th17 immune responses in regulating immune reactivity (Chaudhry et al., 2011; Couper et al., 2008; Gu et al., 2008). Berg et al. (1996) has also reported a role for Th1 immune response in driving enterocolitis in IL‐10 KO mice. It is noteworthy that the higher gene expression of these proinflammatory cytokines in the female IL‐10 KO mice corresponds with their reduced *Tgfb* mRNA. TGF‐β impinges on Th1 differentiation by repressing *Tbx21* and *Stat5a*, which encode the master transcription factors for Th1 cells (Gorelik & Flavell, 2000; Lin et al., 2005). The proinflammatory gut signature of the IL‐10 KO mouse could contribute to the loss of epithelial tight junction (Capaldo & Nusrat, 2009; Ma et al., 2004). The repression of *Tjp1* and *Ocln* in male and female IL‐10 KO mice, respectively, at 3 months supports the early onset of inflammaging and further suggests a potential sex‐based difference in the expression of these tight junctions. A compromised gut is critical to the commonly reported increased systemic inflammation in the IL‐10 KO mice (Alake et al., 2023; Kühn et al., 1993).

As anticipated, both the female and male IL‐10 KO mice exhibited a bone phenotype characterized by lower cortical and trabecular bone mass. Relative to the WT control mice, alterations in *Rankl* and *Wnt10b* in the IL‐10 KO mice suggest greater osteoclastogenesis and reduced osteoblastogenesis contributed to this phenotype. Osteocytes are a major source of IL‐6 in the bone which has been reported to be increased in rodent models of IBD (Metzger et al., 2017). The higher *Il6* mRNA noted in the IL‐10 KO mice have the potential to stimulate osteoclast bone resorption (Ishimi et al., 1990; Löwik et al., 1989) and upregulate the expression of the phosphaturic hormone, FGF23 (Durlacher‐Betzer et al., 2018). This response occurs in conjunction with the IL‐10 KO mice's reduced expression of bone *Phex* and *Dmp1*, which encode for PHEX and DMP1 and regulate FGF23 (Ling et al., 2005; Martin et al., 2011). PHEX represses Fgf23 gene expression mainly through the FGF receptor signaling (Bär et al., 2019; Lu & Feng, 2011; Martin et al., 2011). More so, PHEX and DMP1 are interaction partners and evidence support the importance of their interaction in lowering plasma FGF23 (Martin et al., 2012). Importantly, the increased expression of osteopontin and osteocalcin in the bone tissue also contributes to the bone phenotype in the IL‐10 KO mice. Osteocalcin appears to facilitate bone resorption (Ducy et al., 1996), whereas osteopontin is a substrate for PHEX and it is known for inhibiting bone mineralization (Barros et al., 2013). The reduced PHEX expression in the IL‐10 KO mice therefore explains the increase in *Opn* expression and the reduced bone phenotype in IL‐10 KO mice.

The increased cardiac fibrosis in IL‐10 KO mice at 6 months could be partly attributed to an increase FGF23 in these mice. Egli‐Spichtig et al. (2019) had previously reported an increased serum iFGF23 in IL‐10 KO mice. Higher serum FGF23 had also been reported to mediate cardiovascular pathologies, including left ventricular hypertrophy, cardiac fibrosis, and vascular calcification (Böckmann et al., 2019; Faul et al., 2011; Hao et al., 2016; Jimbo et al., 2014). Furthermore, we reported a reduced E2 signaling in the cardiac tissue of KO mice, as indicated by a reduced phosphorylation of NOS3 as well as a reduced PI3K expression in the cardiac tissue of the IL10 KO mice. Haynes et al. (2000) had earlier demonstrated that the E2 activation of NOS3 occurs via the PI3K‐Akt pathway and it involves the phosphorylation and activation of Ser^1119^ residue of NOS3. More so, the anti‐inflammatory potential of E2 was reported to be mediated by an increased IκBα and the prevention of NFκB nuclear translocation or binding (Ghisletti et al., 2005; Xing et al., 2012). In addition, an increased Ser^536^ phosphorylation of NFκB reportedly inhibits p65 signaling and inflammation (Pradère et al., 2016). Our findings, including the reduced expression of IκBα and phospho Ser^536^ NFκB in the cardiac tissue of IL‐10 KO mice, therefore support a reduced activation of NOS3 and increased inflammation due to reduced E2 signaling in the cardiac tissue of these mice.

In this study, we also demonstrated that IL‐10 KO mice had lower circulating 17‐ β estradiol. 17‐ β estradiol mediates an anti‐inflammatory response in various tissues, including the gut, bone, and vascular endothelium (Goodman et al., 2020; Nilsson, 2007; Weitzmann & Pacifici, 2006). Therefore, the increased gene expression of IL‐6 in the bone and cardiac tissue could be partly attributed to estrogen deficiency in the IL‐10 KO mice. The lowered serum 17‐β estradiol in these mice is explained in part by reduced estrogen synthesis in the ovaries and in adipose tissue as evidenced by a reduced ovarian and WAT *Cyp17a1* and *Cyp11a1* mRNA. These genes encode for two cytochrome P450 enzymes, both catalyzing important steps in the conversion of cholesterol to estrogens. However, recycling of estrogen was also altered in the KO mice as indicated by reduced activity of microbial β‐glucuronidase. β‐glucuronidase increases serum estrogen by deconjugating the glucuronide‐complex and increasing the bioavailability of estrogen (Baker et al., 2017). Interestingly, *Lactobacillus rhamnosus*, which has β‐glucuronidase activity (Biernat et al., 2019) had been reported to prevent bone loss and reduce intestinal inflammation and gut permeability in sex‐steroid‐deficient mice (Li et al., 2016). However, our finding contradicted a phylogenetic prediction of an increased β‐glucuronidase in female IL‐10 KO mice that was earlier reported by Son et al. (2019) using the Phylogenetic Investigation of Communities by Reconstruction of Unobserved States (PICRUSt) software. This difference might be due to the incomprehensive nature of the 16S sequencing data that was used in those predictions. Moreover, such predictions are gene‐level based suggesting the transcriptional, posttranscriptional and translational regulation of gut bacterial β‐glucuronidase warrants further investigation.

The estrogen receptor, ESR2 mediates the anti‐inflammatory role of E2 as well as its role in intestinal organoid regeneration (Goodman et al., 2020; Hases et al., 2020; Lee et al., 2019). Our result showed that E2 treatment does not affect the proinflammatory phenotype in isolated lymphocytes from IL‐10 KO mice. However, E2 treatment increased the transcription of tight junctions in intestinal organoids. These findings suggest an interference of E2 signaling by other factors that are present in an inflamed gut. Interestingly, Pierdominici et al. (2015) had previously reported significant repression of *Esr2* mRNA in T lymphocytes and intestinal epithelial cells exposed to IL‐6. More so, the role of IL‐10 in suppressing IL‐6 production has been previously reported (Hempel et al., 1995). Therefore, the lack of IL‐10 favored the dominance of proinflammatory molecules that could act partially by suppressing the expression of estrogen receptors to increase proinflammatory response in the gut of IL‐10 KO mice.

There are a few limitations in this study. First, the utilization of a global KO model makes it seemingly impossible to fully understand the tissue‐specific role of IL‐10. Future experiments should consider utilizing tissue‐specific KO models to understand the specific roles of IL‐10 in the tissues that were studied and how IL‐10 affects interorgan communication in these tissues. The gene expression of tight junctions does not necessarily determine their localization pattern; however, we are unable to confirm the localization pattern in the tissues that are available. Even though no differences were detected in Tjp1 and Ocln gene expression, future studies should include immunostaining techniques to determine if localization patterns are altered in the IL‐10KO mice since these changes can occur without alterations in overall gene expression. Our supporting in vitro experiments on the effect of E2 in LP lymphocytes and intestinal organoid was limited to female retired breeders. It will be interesting to perform similar experiment at a younger age in both male and female mice. In addition, since lowered E2 was a serendipitous finding at the end of the study and only retired breeders were available, we could not compare the expression level of *Cyp2a1* and *Cyp2a11* for the age group of mice that were studied. Lastly, whereas it is tempting to assume that E2 was lowered due to the deficiency in IL10, further research is needed to understand the mechanism by which reduced recycling and synthesis of E2 occurs in the IL‐10 KO mice.

Based on our findings, including a gut proinflammatory phenotype, an increased bone *Fgf23* with corresponding reduced *Dmp1* and *Phex* mRNA, a reduced serum E2 and gut bacterial β‐glucuronidase, as well as a reduced expression of estrogen receptor or downstream target of E2, we conclude that it is likely the combination of reduced IL‐10 and E2 signaling that promote an inflammatory phenotype which increases the secretion of FGF23. As a result, both factors mediate the interorgan communication that leads to gut inflammation and compromised barrier integrity, cardiac dysfunction and decrements in bone mass in the IL‐10 KO mouse.

## AUTHOR CONTRIBUTIONS

Edralin A. Lucas and Brenda J. Smith funding acquisition; Sanmi E. Alake, Karen Wozniak, Dingbo Lin, Winyoo Chowanadisai, Brenda J. Smith, and Edralin A. Lucas designed research; Sanmi E. Alake, John Ice, Kara Robinson, Payton Price, Bethany Hatter, Brenda J. Smith, and Edralin A. Lucas conducted research; Sanmi E. Alake, Brenda J. Smith and Edralin A. Lucas analyzed the data, wrote the paper, and had primary responsibility for the final content. All authors read and approved the final manuscript.

## FUNDING INFORMATION

This study was funded by the Oklahoma Agricultural Experiment Station (Project # OKL03104 and OKL03105) and the Jim and Lynn Williams Professorship (1‐156560).

## CONFLICT OF INTEREST STATEMENT

All authors declared no conflicts of interest.

## ETHICS STATEMENT

All procedures were approved and followed strict guidelines set by the Institution Animal Care of Oklahoma State University (Protocol #IACUC 22‐02‐STW).

## Supporting information


Data S1.
