# Supplementary material for: Reduced estrogen signaling contributes to bone loss and cardiac dysfunction in interleukin‐10 knockout mice
Source: Physiol Rep. 2024 Jan 12;12(1):e15914. doi: 10.14814/phy2.15914 (PMC10787104; doi:10.14814/phy2.15914)
Supplement: Supplementary file 1 — Data S1. [file PHY2-12-e15914-s001.docx]

Reduced Estrogen Signaling Contributes to Bone Loss and Cardiac Dysfunction in Interleukin-10 Knockout Mice

SE Alake

**Supplemental Table 1:** Primer Sequence for Gene Expression Analyses

| Gene | Forward (5’🡪 3’) | Reverse (5’🡪 3’) |
| --- | --- | --- |
| *Adgre1* | ggcctgtctgcatgatcatca | agcaacctcgtgtccttgagt |
| *Alpi* | aggacatcgccactcaactc | ggttccagactggttactgtca |
| *Arg1* | cagtctggcagttggaagca | gcatccacccaaatgacaca |
| *Bglap2* | tgagcttaaccctgcttgtgacga | agggcagcacaggtcctaaatagt |
| *Col1a1* | cgtctggtttggagagagcat | Ggtcagctggataccgacatc |
| *Cyp11a1* | cagttggttccactcctcaaagc | gaagcaccaggtcattcacagtg |
| *Cyp19a1* | tgagacacatcatgctggacacc | ggtttgatgaggagagcttgcca |
| *Dmp1* | ctgtcattctccttgtgttcct | caaatcacccgtcctctcttc |
| *Esr1* | gcagatagggagctggttca | tggagattcaagtccccaaa |
| *Esr2* | gccaacctcctgatgcttct | tcgtacaccgggaccacat |
| *Fgf23* | cccccatcagaccatctaca | ttcgagtcatggctcctgtt |
| *Gapdh* | caaggtcatccatgacaactttg | ggccatccacagtcttctgg |
| *Icam1* | ggaggtggcgggaaagtt | tccagccgaggaccatacag |
| *Il17a* | ccagggagagcttcatctgt | aggaagtccttggcctccgt |
| *Il1b* | caaccaacaagtgatattctccatg | gatccacactctccagctgca |
| *Il6* | gaggataccactcccaacagacc | aagtgcatcatcgttgttcataca |
| *Ocln* | acccgaagaaagatggatcg | catagtcagatgggggtgga |
| *Opg* | gttcttgcacagcttcacca | aaacagcccagtaccattc |
| *Opn* | actccaatcgtccctacactcg | tgaggtcctcatctgtggcat |
| *Phex* | ggcatgactgctgtaagatcagat | agctccattgacataaggcact |
| *RankL* | tctgcagcatcgctctgttc | agcagtgagtgctgtcttctgatatt |
| *Sost* | accgggcggagaatgg | gctgtactcggacacatctttgg |
| *Tgfb* | ctcccgtggcttctagtgc | gccttagtttggacaggatctg |
| *Tjp1* | agactggttgtttaggagca | cagaatacggctccttcctg |
| *Tnf* | ctgaggtcaatctgcccaagtac | cttcacagagcaatgactccaaag |
| *Vcam1* | tgaacccaaacagaggcagagt | ggtatcccatcacttgagcagg |
| *Vil1* | tcaaaggctctctcaacatcac | agcagtcaccatcgaagaagc |
| *Wnt10b* | atgcggatccacaacaacag | ttccatggcatttgcacttc |

**Supplemental Figure 1:** Food intake and bone mineral content (BMC) of interleukin (IL)-10 knock-out (KO) and wild type (WT) mice at 3m and 6m timepoint.


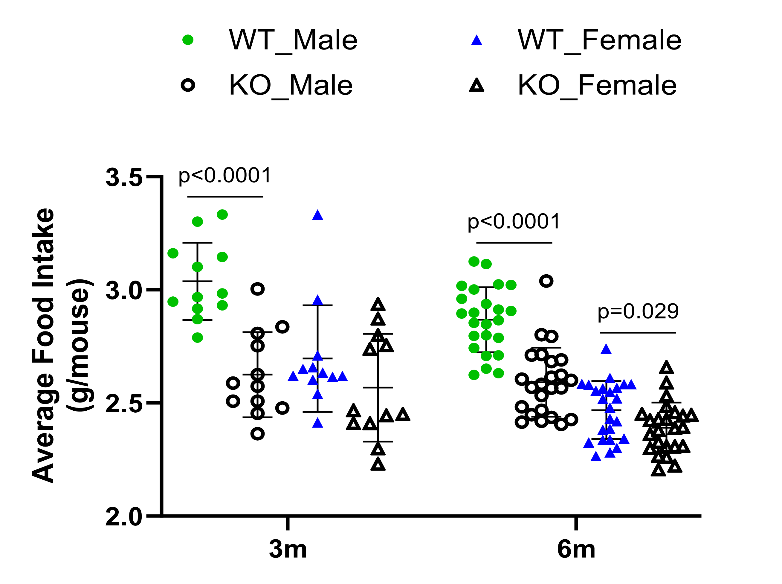

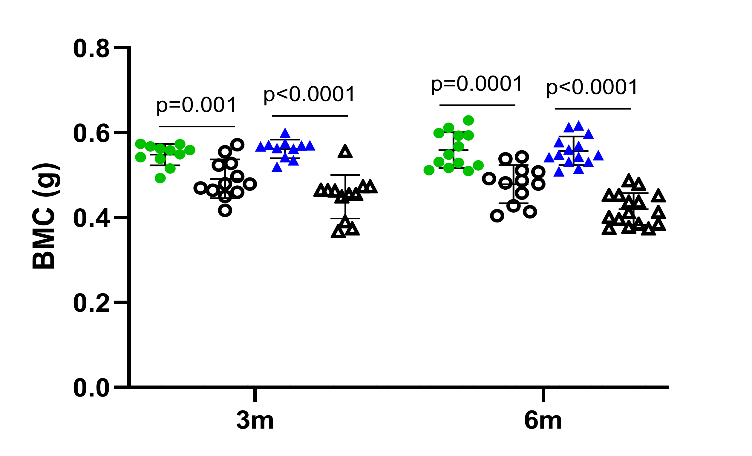

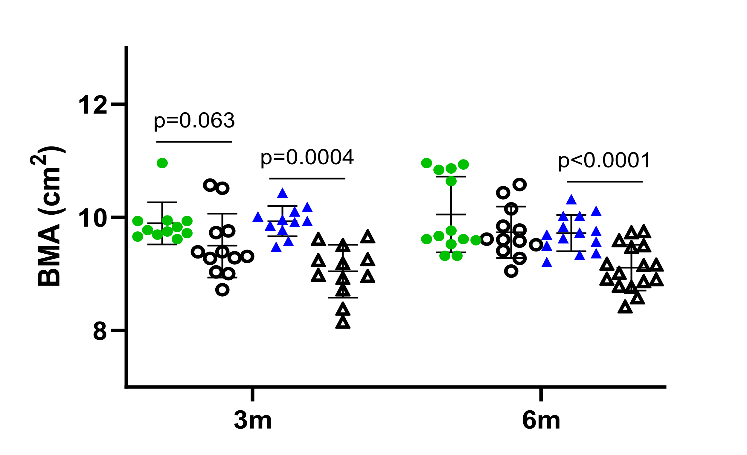


a)

c)

b)

Average food intake (A), bone mineral content, BMC (B), and bone mineral area, BMA (C) in WT mice and KO mice fed a semi-purified diet for 3m or 6m. Values are mean ± SD n=12-16 mice/group for A, n=7-10 mice/group for B, and n=12-16 mice/group for C and D (independent student t test).

**Supplemental Figure 2:** Bone microarchitecture parameters of interleukin (IL)-10 knock-out (KO) and wild type (WT) mice at 3m and 6m timepoint.


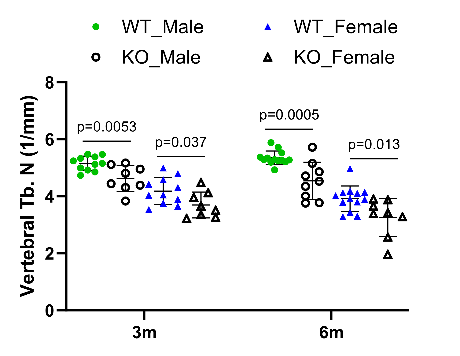

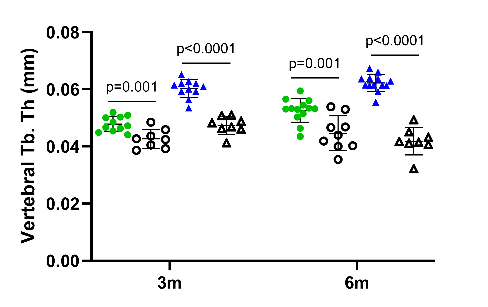

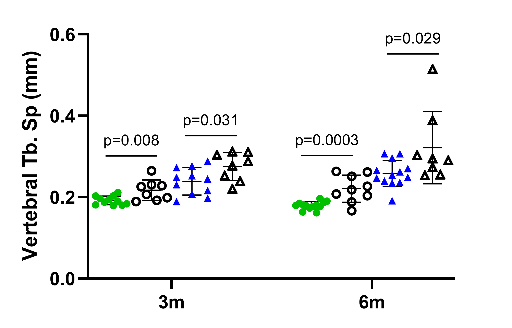

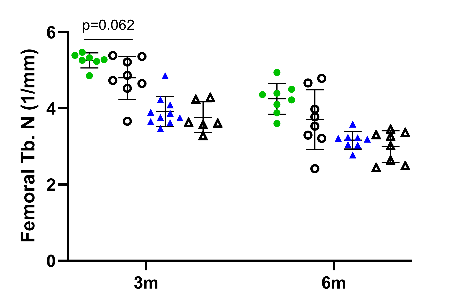

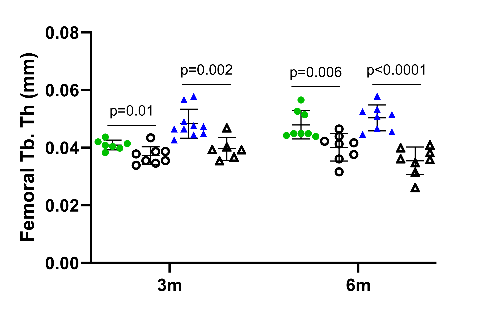

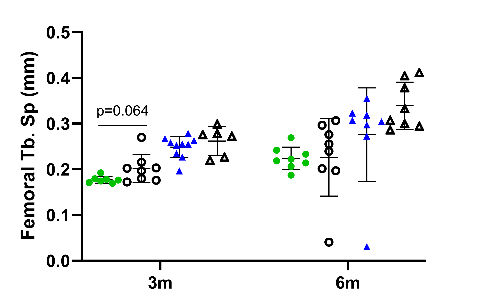

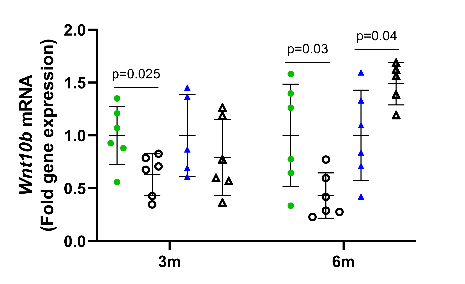

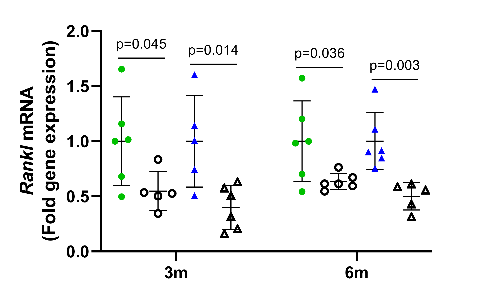

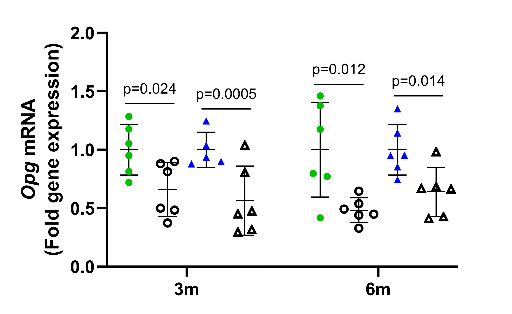


a)

b)

c)

d)

e)

f)

g)

i)

h)

j)


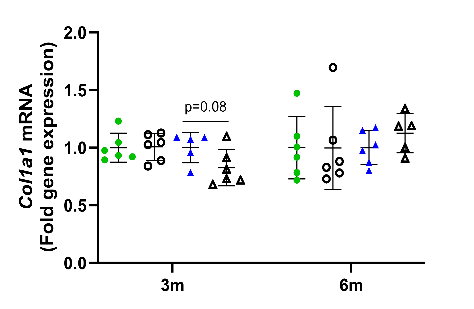


Vertebral: trabecular number, TbN (A), trabecular thickness, TbTh (B), and trabecular separation, TbSp (C). Femoral: trabecular thickness (D), trabecular number (E), and trabecular separation (F). Femur gene expression of *Wnt10b* (G), *Col1a1* (H) *RankL* (I), and *Opg* (J) in WT mice and KO mice fed a semi-purified diet for 3m or 6m. Values are mean ± SD, n=8-13 mice/group for A-F, n=6 mice/group for G-J. (independent student t test).

**Supplemental Figure 3:** Cardiac gene expressions in interleukin (IL)-10 knock-out (KO) and wild type (WT) mice at 3m and 6m timepoints


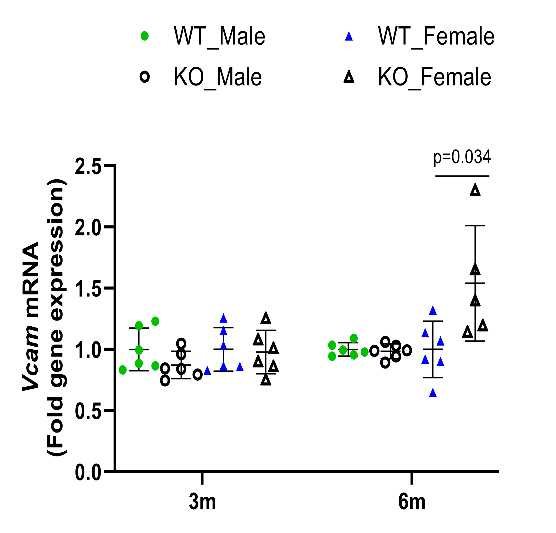

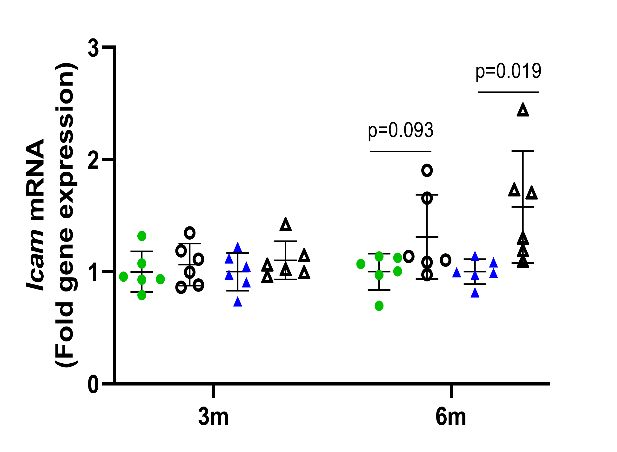

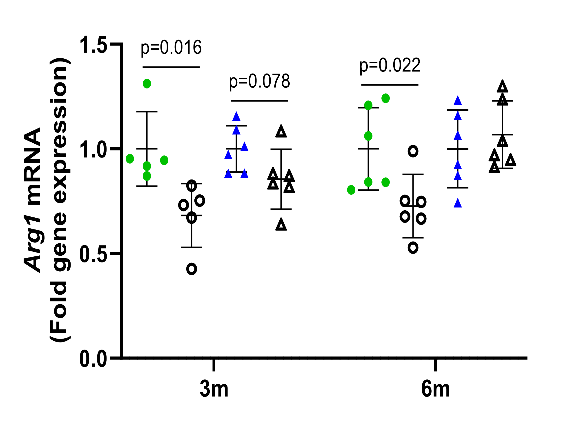


a)

b)

c)

Cardiac gene expression of adhesion molecules or macrophage marker: *Vcam1* (A), *Icam1* (B) and *Arg1* (C) in WT mice and KO mice fed a growth diet for 3m or 6m. Values are mean ± SD, n=6 mice/group. (independent student t test).

**Supplemental Figure 4:** Estrogen-related parameters in interleukin (IL)-10 knock-out (KO) and wild type (WT) mice


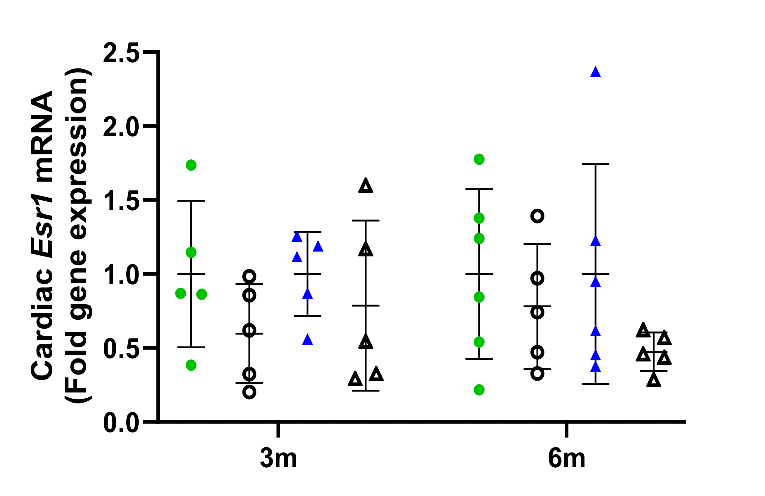

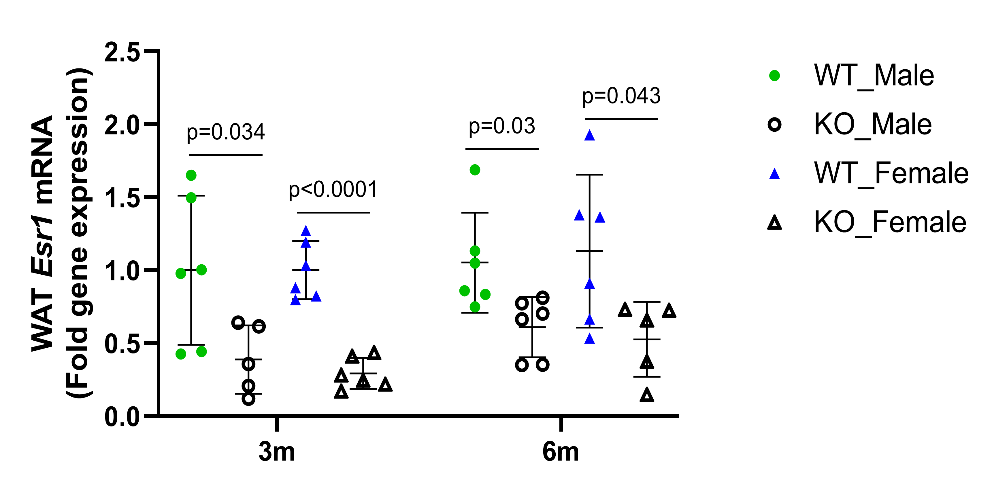


a)

b)

Gene expression of *Esr1* in the cardiac (A) and adipose tissue (B) in WT mice and KO mice fed a semi-purified diet for 3m or 6m. Values are mean ± SD, n=6 mice/group. (independent student t test).

**Supplemental Figure 5:** Gene expression parameters in E2-treated intestinal organoids of interleukin (IL)-10 knock-out (KO) and wild type (WT) mice


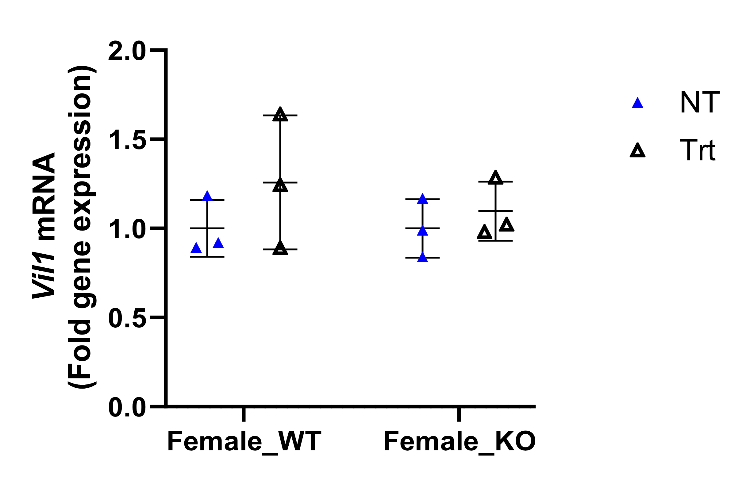

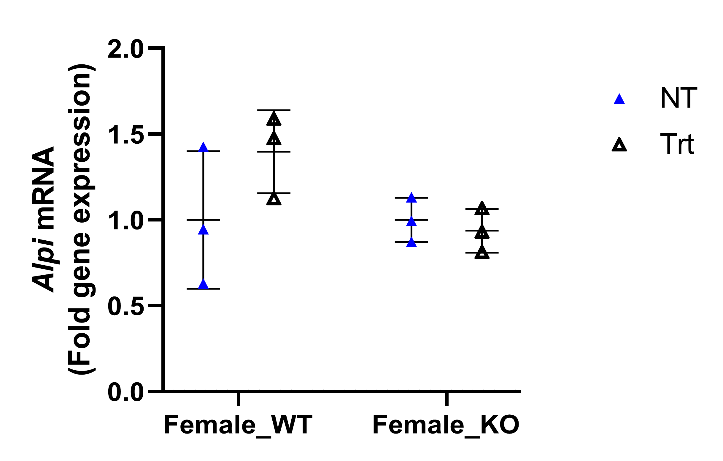


a)

b)

Gene expression of: Vil1 (A), and *Alpi* (B) in E2-treated (100nM) intestinal organoid developed from crypt cells isolated from 14m-old female KO and WT mice. Values are mean ± SD, n=3 mice/group, replicates =3/mouse. Analyzed by independent student t test.
